# Mild SARS-CoV-2 Infection After Gene Therapy in a Child With Wiskott-Aldrich Syndrome: A Case Report

**DOI:** 10.3389/fimmu.2020.603428

**Published:** 2020-11-24

**Authors:** Sabina Cenciarelli, Valeria Calbi, Federica Barzaghi, Maria Ester Bernardo, Chiara Oltolini, Maddalena Migliavacca, Vera Gallo, Francesca Tucci, Federico Fraschetta, Elena Albertazzi, Elena Sophia Fratini, Giulia Consiglieri, Stefania Giannelli, Francesca Dionisio, Claudia Sartirana, Sara Racca, Chiara Camesasca, Giovanni Peretto, Rita Daverio, Antonio Esposito, Francesco De Cobelli, Paolo Silvani, Marco Rabusin, Andrea Cara, Daria Trabattoni, Stefania Dispinseri, Gabriella Scarlatti, Lorenzo Piemonti, Vito Lampasona, Maria Pia Cicalese, Alessandro Aiuti, Francesca Ferrua

**Affiliations:** ^1^ Pediatric Immunohematology and Bone Marrow Transplantation Unit, IRCCS San Raffaele Scientific Institute, Milan, Italy; ^2^ Vita-Salute San Raffaele University, Milan, Italy; ^3^ San Raffaele Telethon Institute for Gene Therapy (SR-Tiget), IRCCS San Raffaele Scientific Institute, Milan, Italy; ^4^ Clinic of Infectious Diseases, Division of Immunology, Transplantation and Infectious Diseases, IRCCS San Raffaele Scientific Institute, Milan, Italy; ^5^ Laboratory of Medical Microbiology and Virology, IRCCS San Raffaele Hospital, Milan, Italy; ^6^ Pediatric Cardiology, Cardio-thoraco-vascular Department, IRCCS San Raffaele Scientific Institute, Milan, Italy; ^7^ Myocarditis Unit, Department of Cardiac Electrophysiology and Clinical Arrhythmology, IRCCS San Raffaele Scientific Institute, Milan, Italy; ^8^ Department of Clinical Biochemistry, IRCCS San Raffaele Scientific Institute, Milan, Italy; ^9^ Clinical and Experimental Radiology Unit, Experimental Imaging Center, IRCCS San Raffaele Institute, Milan, Italy; ^10^ Department of Anesthesia and Critical Care, IRCCS San Raffaele Scientific Institute, Milan, Italy; ^11^ Department of Pediatrics, HematoOncology Unit, Institute of Maternal and Child Health Burlo Garofolo, Trieste, Italy; ^12^ National Center for Global Health, Istituto Superiore di Sanità, Rome, Italy; ^13^ Department of Biomedical and Clinical Sciences “L. Sacco”, University of Milan, Milan, Italy; ^14^ Viral Evolution and Transmission Unit, IRCCS San Raffaele Scientific Institute, Milan, Italy; ^15^ Beta Cell Biology Unit, Diabetes Research Institute, IRCCS San Raffaele Scientific Institute, Milan, Italy; ^16^ Division of Genetics and Cell Biology, IRCCS San Raffaele Scientific Institute, Milan, Italy

**Keywords:** Wiskott-Aldrich Syndrome, primary immunodeficiencies, gene therapy, immune reconstitution, severe acute respiratory syndrome Coronavirus 2 (2019-nCoV)

## Abstract

In this work we present the case of SARS-CoV-2 infection in a 1.5-year-old boy affected by severe Wiskott-Aldrich Syndrome with previous history of autoinflammatory disease, occurring 5 months after treatment with gene therapy. Before SARS-CoV-2 infection, the patient had obtained engraftment of gene corrected cells, resulting in WASP expression restoration and early immune reconstitution. The patient produced specific immunoglobulins to SARS-CoV-2 at high titer with neutralizing capacity and experienced a mild course of infection, with limited inflammatory complications, despite pre-gene therapy clinical phenotype.

## Introduction

Data about severe acute respiratory syndrome Coronavirus 2 (SARS-CoV-2) infection in children with primary immunodeficiencies (PIDs) are limited (1). In the general pediatric population, this infection is known to be generally milder than in adults (2, 3). However, multisystem inflammatory syndrome, temporally associated to SARS-CoV-2 infection, has been increasingly reported in children and adolescents. Clinical spectrum ranges from general inflammatory syndromes to incomplete and complete forms of Kawasaki-like disease, leading to serious illness with wide cardiovascular involvement, suggestive of a systemic immune-mediated disease (4, 5).

Here we present the first case of a 1.5-year-old boy affected by severe Wiskott-Aldrich Syndrome (WAS), who experienced SARS-CoV-2 infection five months after treatment with gene therapy (GT). WAS is a rare, X-linked, life-threatening PID, caused by mutations in the gene encoding for the WAS protein (WASP), a key regulator of actin polymerization. WASP deficiency in platelets results in micro-thrombocytopenia, while in immune cells it mainly compromises immunological synapse formation, cell migration and cytotoxicity. Thus, WAS is characterized by bleeding episodes, development of recurrent or severe infections, eczema and increased risk of autoimmunity, autoinflammation and malignancies (6). Supportive treatment is based on immunogloblulin replacement therapy, antimicrobial prophylaxis and immunosuppressants. Allogeneic hematopoietic stem/progenitor cell (HSPC) transplantation is a recognized curative treatment for WAS, even if it may be hampered by complications such as graft-versus-host disease, rejection and autoimmunity. Moreover, donor availability may be limited. Investigational autologous gene therapy (GT) represents a safe and effective therapeutic alternative, according to available data from recent GT clinical trials using lentiviral vectors encoding for the human *WAS* gene (7–10).

## Case Presentation

Our patient was diagnosed with WAS (*WAS* gene mutation: c.1384_1385delAG; p.S461Lfs*32) at 3 months of age, due to severe thrombocytopenia, eczema and early-onset steroid-refractory autoinflammatory manifestations (fever, vasculitis, increased inflammatory indexes, Zhu score 5A), treated with IL-1 soluble receptor antagonist. After diagnosis, anti-infective prophylaxis and immunoglobulin replacement therapy were also started. At 5 months of age, he experienced severe hypereosinophilia with mild cardiac injury, which required treatment with steroids. In the following months, he also developed chronic CMV infection with multiple reactivations, requiring specific antiviral treatment.

**Figure 1:**
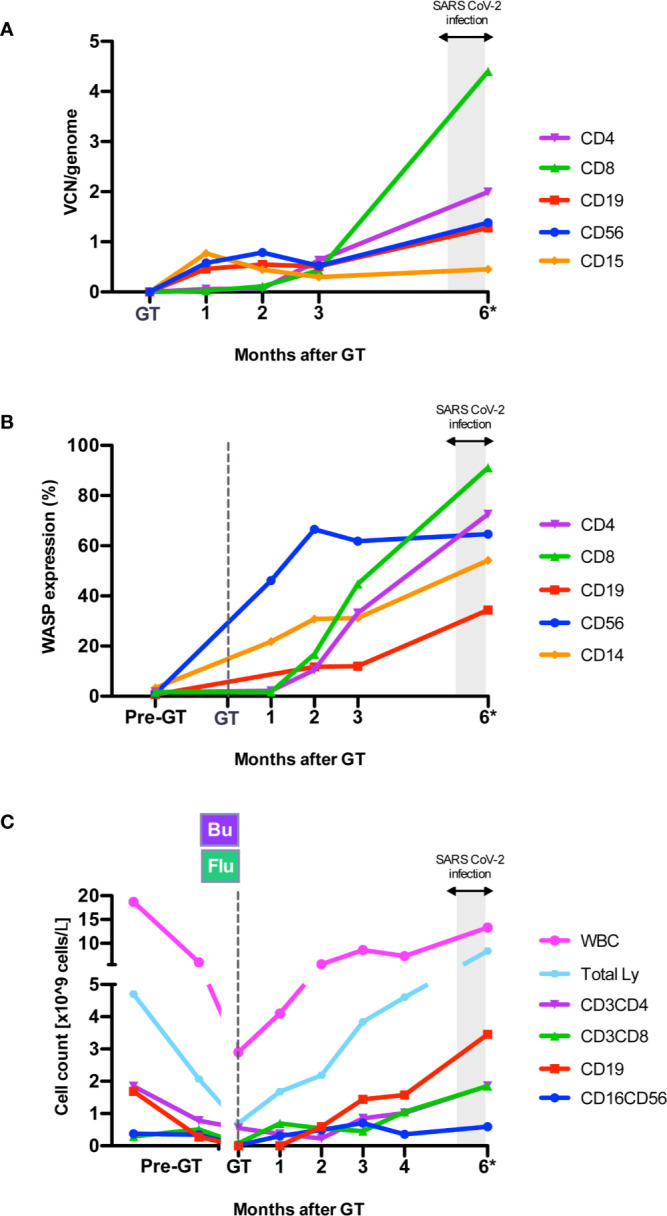
Immune reconstitution after gene therapy (GT). **(A)** Engraftment of gene corrected cells expressed as VCN/genome in sorted subpopulations from peripheral blood (PB), measured by Real Time-PCR (10) during follow-up after GT. VCN, Vector Copy Number. **(B)** WASP expression (% of WASP+ cells) by flow cytometry (10) in PB cell subpopulations. WASP, WAS protein. **(C)** Peripheral blood cell counts at different time points before GT and during follow-up. Bu, Busulfan; Flu, fludarabine. *The 6 month-follow up visit was performed between 6.4 and 6.9 months after GT, after second negative swab for SARS-CoV2.

In 2019, at 1 year of age, the patient underwent at our Unit a reduced-intensity conditioning with mAb anti-CD20 (rituximab, 375 mg/sqm), busulfan [weight and area under the curve (AUC)-targeted, target AUC 48,000 ± 10% ng/ml*h] and fludarabine (total dose: 60 mg/sqm), followed by GT with OTL-103 [autologous CD34+ cell-enriched population containing HSPC transduced *ex vivo* using a lentiviral vector encoding the human *WAS* gene] in a clinical trial (OTL-103-4; EudraCT number: 2018-003842-18; NCT03837483) (10). Sinusoidal obstruction syndrome with suspected thrombotic microangiopathy occurred early after GT, possibly linked to an unexpected high busulfan exposure (AUC 80,988 ng/ml*h), and then resolved without sequelae. Neutrophil engraftment was achieved on day +15 post-GT. Multilineage engraftment of gene corrected cells resulted restoration of WASP expression in lymphocytes and platelets, improvement of *in vitro* T-cell functions, in particular anti-CD3i mediated response (11), and main lymphocyte subsets’ count normalization (T, B and NK cells) by 4 months post-GT (**Figure 1**, **Supplementary Figure 1** and data not shown). At the same timepoint, platelets ranged between 21 and 29 *10^9^/L.

**Figure 2:**
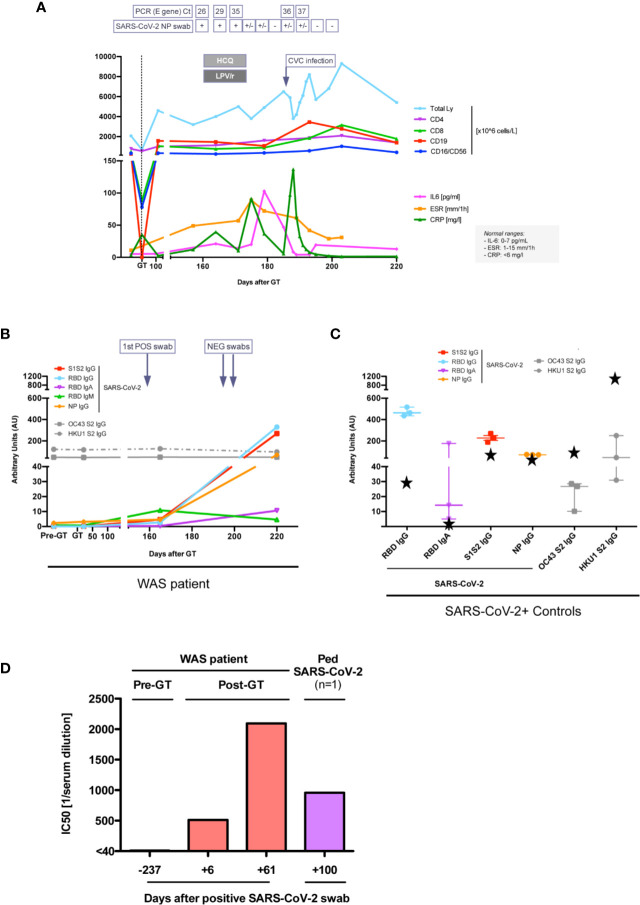
Immune response to SARS-CoV-2 infection. **(A)** Lymphocyte subpopulations counts and inflammatory marker levels during SARS-CoV-2 infection. SARS-CoV-2 E gene real time (RT)-PCR cycle threshold (Ct) on nasopharyngeal (NP) swabs are reported, when available. +, positive; +/−, weakly positive; –, negative. HCQ, hydroxychloroquine; LPV/r, lopinavir/ritonavir. CVC, central venous catheter. Units of measure of data reported on y axis are specified in the legend on the right. **(B, C)** Anti-SARS-CoV-2 and anti-OC43/HKU1 Coronaviruses antibody serum level in our patient **(B)**, in three children and in patient’s mum (black stars) after SARS-CoV-2 infection **(C)**. **(D)** 50% neutralization (IC50) (expressed as reciprocal of serum dilution) of SARS-CoV-2 with the serum of our patient and of another child after SARS-CoV-2 infection. The first dilution tested was 1/40.

In March 2020 (5 months after GT), our patient tested positive by RT-PCR for SARS-CoV-2 at nasopharyngeal and rectal swabs after his mildly symptomatic mother tested positive (**Figure 2A**). Although the patient was asymptomatic, due to the high risk of SARS-CoV-2-related complications based on his previous clinical history, *off-label* home-treatment with hydroxychloroquine (HCQ, 2.5 mg/kg BID) and lopinavir/ritonavir (LPV/r, 12 mg/kg BID) was administered for 2 weeks. Blood tests and nasopharyngeal swabs were monitored weekly, showing mild increase in inflammatory indexes (**Figure 2A**), without lymphopenia. During infection period platelets ranged between 21 and 55 *10^9^/L (patient on ongoing TPO-agonist treatment) and showed normal volume, as expected based on previous studies (7, 12). There was no evidence of pro-coagulant status, hyperferritinemia or dyslipidemia. Nasopharyngeal swab RT-PCR became weakly positive 16 days after the first positivity detection, and two consecutive negative nasopharyngeal swabs were obtained within a total of 40 days.

One month after the first SARS-CoV-2 finding, the patient experienced a concomitant central venous catheter (CVC)-related infection from Staphylococcus epidermidis with fever and an increase in inflammatory indexes, which rapidly resolved with antibiotic treatment and CVC removal. At this time, repolarization anomalies at electrocardiogram (ECG) and increase of cardiac biomarkers (troponin T, pro-BNP, CK-MB) suggested ongoing myocardial inflammation (**Supplementary Table 1**). Thus, high-dose intravenous immunoglobulins (IVIg) (1 g/kg), but not steroids, were administered. Notably, transthoracic echocardiogram and 24-h Holter ECG were unremarkable. Furthermore, completely normal findings at delayed-enhanced cardiac magnetic resonance allowed to rule out myocarditis.

Specific antibodies to different SARS-CoV2 antigens were tested by a luciferase immunoprecipitation system (LIPS) assay developed in house (13). Serum IgG to Nucleocapsid protein (NP), Receptor binding domain (RBD) and Spike proteins (S1S2) were positive by day 6 after first positive swab and at day 61 (**Figure 2B**), reaching levels comparable to those of children who experienced SARS-CoV2 infection (n=3) and higher than the mother (**Figure 2C**). Serum IgA and IgM to RBD were also detected in the patient. Notably, at all time points the patient had a stable positive signal for antibodies against two coronaviruses associated with common cold (HKU1 and OC43) (**Figure 2B**). Since the patient was still on monthly IVIg supplementation, we tested eight children receiving Ig supplementation, all with negative SARS-CoV-2 swabs, and followed at our Unit in the same period. They all resulted negative for specific SARS-CoV2 antibodies, indicating their absence in the immunoglobulin preparations and the lack of interference with the LIPS assay (data not shown). Remarkably, the patient’s serum also showed a neutralizing capacity to SARS-CoV2 using a Spike-pseudotyped lentiviral vector in a VERO E6 cell assay (**Figure 2D**) (*manuscript in preparation*). After complete resolution of SARS-CoV-2 infection, molecular and immunological tests showed persistence of engraftment of gene corrected cells, with marked rise in CD8+ transduced cells, increase in WASP-expressing cells and stable lymphocyte count (**Figure 1**). IL-6 increased transiently during acute SARS-CoV-2 infection and then returned to normal values (**Figure 1A**). As compared to pre-GT values, plasma TNF-alpha and IL-1beta as well as cytokines which have been associated with symptomatic Coronavirus Disease 19 (COVID-19) (14) (IL-2, IL-7, IL-10, IFN-g, IL-8) were stable or decreased post-SARS-Cov2 infection. IL-1-alpha, eotaxin and VEGF were increased (data not shown).

## Discussion

SARS-CoV-2 infection is known to cause a wide-spectrum of clinical presentations in adult population, varying from asymptomatic course to severe hyper inflammatory syndromes requiring intensive care (15). It is known that children generally have a milder course, possibly due to a different immunological status or different distribution of receptors compared to adults (3). Outbreaks of Kawasaki and Kawasaki-like syndromes have been described during SARS-CoV-2 pandemic (5), but the pathogenesis and predisposing factors have not yet been identified.

At present, little is known about SARS-CoV-2 infection in children with PID. A recent retrospective survey that included this patient (16) indicates that risk factors predisposing to severe disease and mortality in patients with congenital immune disorders were similar to the general population, but a significant number of younger patients were affected. In a small series in transplant centers in Spain, three immunodeficient patients with SARS-CoV-2 infection were reported post-transplantation, suggesting an increased risk for these patients (17).

WAS is a PID associated with micro-thrombocytopenia, recurrent or severe infections, eczema and increased risk to develop autoimmune and autoinflammatory manifestations, which represented the main clinical features of our patient at disease onset (7). While antiviral treatment (HCQ + LPV/r) was started precociously in our patient, it was not sufficient to induce SARS-CoV-2 viral clearance, with specific swabs remaining positive for 40 days, at the higher range of asymptomatic patients (14).

Although no data are available on early antiviral treatment in asymptomatic/mildly symptomatic subjects, recent work has shown lack of efficacy of HCQ and LPV/r in hospitalized adult with COVID-19 (18, 19).

## Concluding Remarks

Immune reconstitution after GT allowed the patient to present a mild clinical course after infection with SARS-CoV-2, as observed in most children. He did not show lymphopenia, known to associate with longer duration of viremia (15), and controlled the infection with early development of immunity with specific antibody response to viral antigens and neutralizing activity. It is unlikely that the limited inflammatory complications, despite its underlying auto-inflammatory disease, is due to an incomplete immune-hematological reconstitution since at the time of presumptive SARS-CoV2 infection the patient had normal leukocyte counts and subsequently produced a prompt inflammatory response to the concomitant bacterial infection. Although we cannot rule out that our patient experienced a very mild form of COVID-19 related inflammatory syndrome (20), his cardiac biomarkers showed only a mild increase (**Supplementary Table 1**). In addition, he did not fall into the criteria of the Multisystem Inflammatory Syndrome of Children (MISC) and he was discharged after resolution of the bacterial infection. In conclusion, despite previous clinical history of inflammatory manifestations, early immune reconstitution after GT favored the development of specific, neutralizing IgG antibodies against SARS-CoV-2 and mild SARS-CoV-2 disease course without severe autoinflammatory complications.

## Data Availability Statement

The raw data supporting the conclusions of this article will be made available by the authors, without undue reservation.

## Ethics Statement

For the assessment of antibody response to SARS-CoV-2 serum level and neutralization capacity and cytokine analysis, we obtained peripheral blood samples from our patient and healthy controls, in accordance with the Declaration of Helsinki. Written informed consent forms were approved by the institutional ethics committee of the San Raffaele Hospital (Tiget06, Tiget09, ImmCOVID-19 and GENE-COVID protocols). Gene therapy protocol (OTL-103-4, NCT03837483) and other trial-related materials were approved by the independent ethics committee of the San Raffaele Scientific Institute and the Italian regulatory authority (Agenzia Italiana del Farmaco [AIFA]). Written informed consent was provided by patient's parents before initiation of study specific procedures.

## Author Contributions 

SC is an OTL-103-4 clinical trial investigator, provided clinical care for the patient, participated to data collection and analysis and wrote the manuscript. VC, FB, MB, CO, MM, VG, FT, FFr, EF, GC, PS, and MR provided clinical care for the patient. EA contributed to data collection. SG, FD, and CS performed molecular and immunological studies on patient’s samples. SR performed RT-PCR for SARS-CoV-2 on patient’s swabs. CC, GP, AE, and FC were involved in the cardiological assessments and data interpretation. RD performed inflammatory marker and cytokine analyses. AC, SD, and GS developed and performed SARS-CoV-2 neutralization assay. DT performed cytokine profiling experiments. LP and VL developed and performed an assay for the detection of specific antibodies to different SARS-CoV2 antigens. MC is an OTL-103-4 clinical trial investigator, contributed to patient’s care and data interpretation. AA is the OTL-103-4 clinical trial principal investigator, designed the study and contributed to data interpretation and manuscript writing. FFe is an OTL-103-4 clinical trial investigator, coordinated data collection, analysis and interpretation, provided clinical care for the patient, and wrote the manuscript. All authors contributed to the article and approved the submitted version.

## Funding

Supported by Fondazione Telethon, European Community (E-rare EUROCID), and Program Project COVID-19 OSR-UniSR. Study NCT03837483 is sponsored by Orchard Therapeutics (OTL).

## Conflict of Interest

The San Raffaele Telethon Institute for Gene Therapy (SR-Tiget) is a joint venture between the Italian Telethon Foundation and Ospedale San Raffaele (OSR). WAS gene therapy was licensed to GlaxoSmithKline in 2014 and then transferred to Orchard Therapeutics (OTL) in April 2018. AA, FFe, MC and SC are investigators of trial # NCT03837483.

The remaining authors declare that the research was conducted in the absence of any commercial or financial relationships that could be construed as a potential conflict of interest.

The handling editor declared a past co-authorship with several of the authors VG, FB, MC, FFe, and AA.
