# Supplementary material for: Mild SARS-CoV-2 Infection After Gene Therapy in a Child With Wiskott-Aldrich Syndrome: A Case Report
Source: Front Immunol. 2020 Nov 24;11:603428. doi: 10.3389/fimmu.2020.603428 (PMC7732473; doi:10.3389/fimmu.2020.603428)
Supplement: Supplementary file 1 [file DataSheet_1.docx]

**Supplementary Table 1**

**Cardiac Biomarkers values**

|  | Troponin T (ng/L) | proBNP (pg/mL) | CK-MB (µg/L) |
| --- | --- | --- | --- |
| 23.04.2020 | 9.7 | **596** | n.d. |
| 24.04.2020 | 6.4 | **701** | n.d. |
| 27.04.2020 | **15.1** | **230** | n.d. |
| 28.04.2020 | **17.2** | **162** | n.d. |
| 29.04.2020 | 8.2 | **333** | n.d. |
| 30.04.2020 | 10.5 | **249** | n.d. |
| 04.05.2020 | 9.7 | **291** | n.d. |
| 08.05.2020 | 13.8 | **179** | **7.2** |
| 11.05.2020 | 12.7 | **245** | **7.6** |
| 14.05.2020 | **14.7** | **162** | **8** |
| 18.05.2020 | **14.3** | **318** | 6.1 |
| 25.05.2020 | **17.7** | **249** | **7.5** |

Normal values:

Troponin T: <14 ng/L

ProBNP: 0-88 pg/mL

CK-MB: 0.4-7.0 µg/L

**Figure legend**

**Supplementary Figure 1.**

**(A)** Percentage of platelets expressing WASP, as measured by flow cytometry. **(B)** T-cell proliferation response after incubation with increasing concentrations of anti-CD3i antigen before gene therapy and at 6 months of follow-up. On the left results are reported as count per minute (cpm); on the right results are expressed as stimulation index (S.I.). * The 6 month-follow up visit was performed between 6.4 and 6.9 months after GT, after second negative swab for SARS-CoV2.
