# Supplementary figures and images for: Mild SARS-CoV-2 Infection After Gene Therapy in a Child With Wiskott-Aldrich Syndrome: A Case Report
Source: Front Immunol. 2020 Nov 24;11:603428. doi: 10.3389/fimmu.2020.603428 (PMC7732473; doi:10.3389/fimmu.2020.603428)

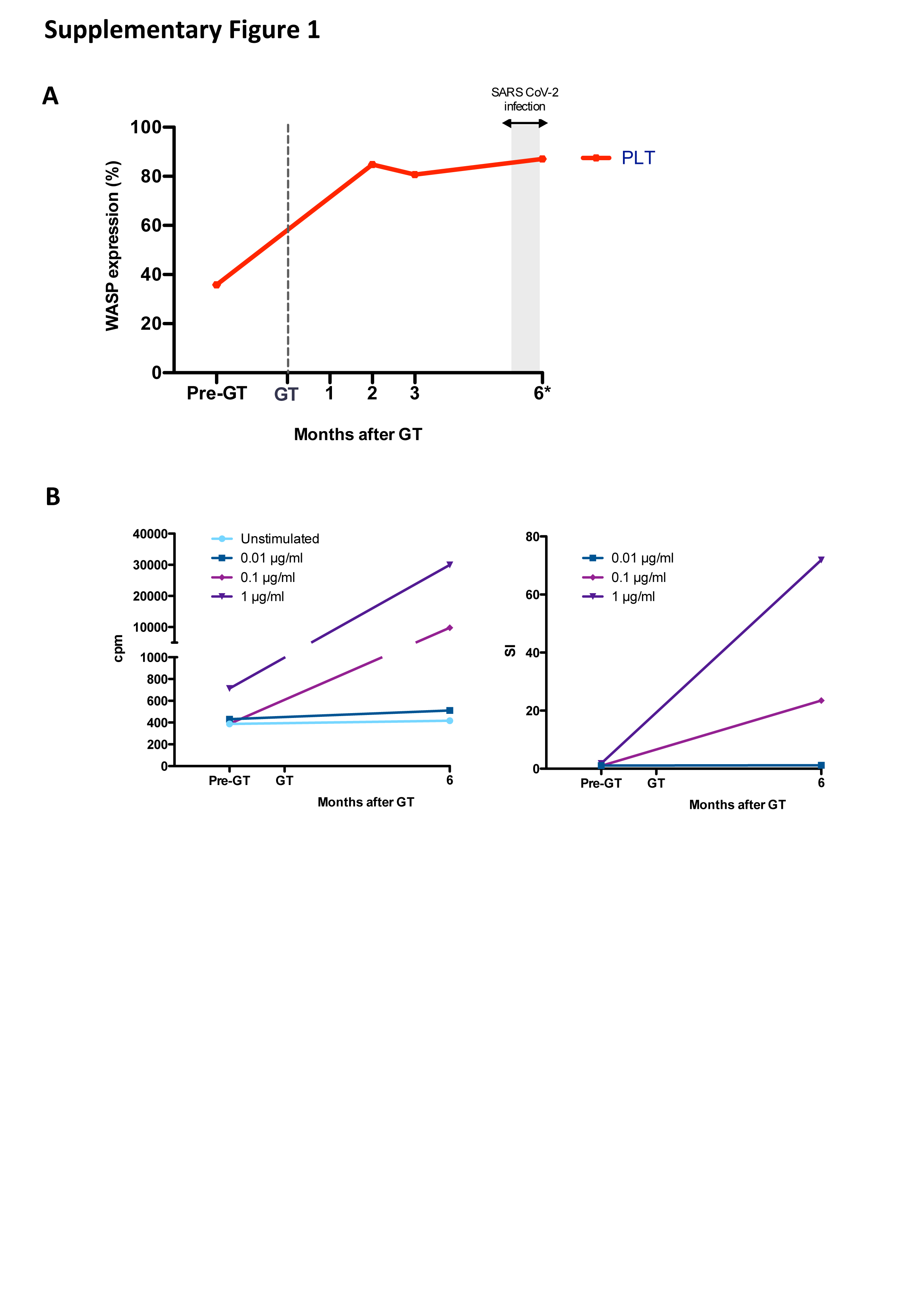

Supplement: Supplementary file 2 [file Image_1.tif]
